# Cardiac lipid droplets differ under pathological and physiological conditions

**DOI:** 10.1016/j.jlr.2025.100920

**Published:** 2025-10-01

**Authors:** Ni-Huiping Son, Sunny Son, Michael Verano, Zhen-Xiu Liu, Waqas Younis, Makenzie Komack, Kelly V. Ruggles, Jana Gjini, Song-Tao Tang, Ainara Gonzalez Cabodevilla, Feng-Xia Liang, Hai-Zhen Wang, Dimitrios Nasias, José O. Alemán, Ira J. Goldberg

**Affiliations:** 1 Division of Endocrinology, Diabetes and Metabolism, New York University Grossman School of Medicine, New York, NY, USA; 2 Division of Precision Medicine, New York University Grossman School of Medicine, New York, NY, USA; 3 Microscopy Core Laboratory, Department of Cell Biology, New York University Grossman School of Medicine, New York, NY, USA; 4 Division of Cardiology, New York University Grossman School of Medicine, New York, NY, USA

**Keywords:** heart failure, lipotoxicity, lipolysis, proteomics, lipidomics, ceramides

## Abstract

Excessive accumulation of lipids within cardiomyocytes can sometimes initiate cardiomyopathy, while in other situations excess lipids do not cause harm. To understand how pathologic and non-pathologic lipid accumulation differ, we isolated lipid droplets (LDs) from two genetically altered mouse lines and from wild-type (WT) mice after an overnight fast. The LDs from MHC-peroxisomal proliferator-activated receptor γ1(MHC-*Pparg1*) transgenic mice were threefold larger than those from either fasted WT or non-cardiomyopathy MHC-diacylglycerol acyl transferase 1 (MHC-*Dgat1*) transgenic mice. Proteomic analysis of the LD-associated membrane proteins (LDAMPs) showed that MHC-*Pparg1* LDs had less perilipin (PLIN). Proteins associated with lipolysis and LD formation (CIDEs and MTP), lipid synthesis, and *Pparg* signaling pathways were increased in MHC-*Pparg1* LDAMPs. Unlike in MHC-*Pparg1*, MHC-*Dgat1* LDAMPs exhibited increased mitochondrial peroxidative proteins with reduced adipose triglyceride lipase (Pnpla2), and Pparg coactivator 1 alpha (Pgc1A). Cardiomyocytes from MHC-*Pparg1* hearts had transmission electron microscopy (TEM) images of ongoing lipolysis and greater amounts of lipolytic proteins. In contrast, images from MHC-*Dgat1* cardiomyocytes showed more lipophagy. Consistent with the proteomic study and EM images, cardiac immunofluorescence staining showed that PLIN5 protein, thought to block LD lipolysis, was markedly reduced with MHC-*Pparg1* overexpression, while hormone-sensitive lipase was increased. The autophagosome marker protein LC3B was increased in MHC-*Dgat1* but not in MHC-*Pparg1* hearts. Potentially toxic lipids like diacylglycerols and ceramides were increased in hearts but not LDs from MHC-*Pparg1* mice. Our data indicate that cardiomyocyte LDs vary in size, composition, and metabolism. Cardiotoxicity was associated with greater LD lipolysis, which we postulate leads to intracellular release of toxic lipids.

## Graphical Abstract

**Figure:**
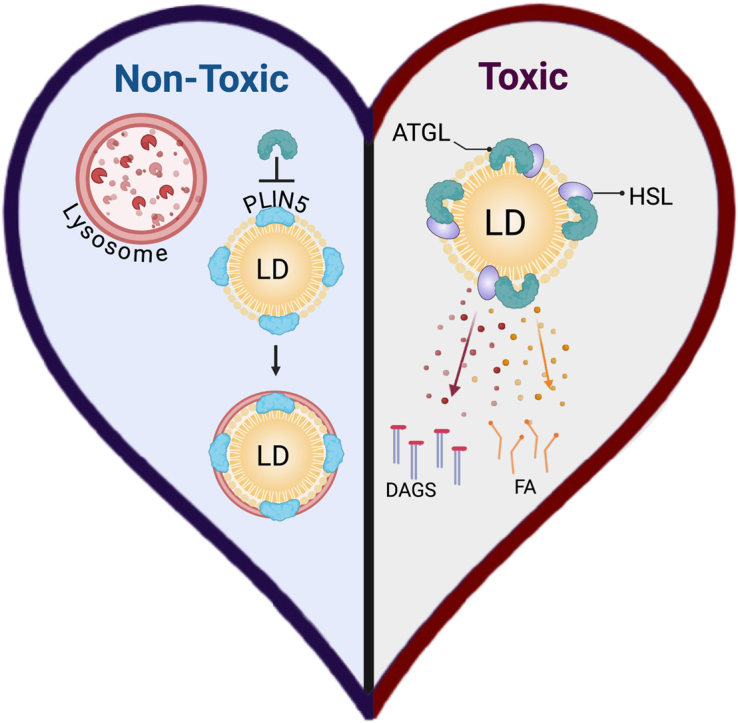

Lipid droplets (LDs) are intracellular and cytoplasmic organelles composed of neutral lipids, mainly triacylglycerols (TAGs) and cholesteryl esters (CE) (1), which are surrounded by a monolayer of phospholipids and associated proteins (2, 3). LDs are involved in multiple cellular processes, such as lipid metabolism (4, 5), protein degradation (6) and nucleic acid processing (7). Lipidomic studies have shown that LDs contain more than 100 different neutral lipids, the fraction of the lipid types varies among different cells and tissues (5, 8, 9, 10). By mass spectrometric analysis, lipid metabolism related enzymes—acyl-CoA synthetases, lipases and sterol biogenesis enzymes—have been identified from LDs of various cell lines and tissues (11, 12, 13, 14, 15). In addition, proteins that are not obviously related to lipids, such as transcription factors, chromatin components, and toxic proteins are isolated with the LDs (16, 17, 18). LDs are not only inner fat depots but also are dynamic as they are synthesized and broken down in response to cellular needs. Moreover, LDs act as hubs that coordinate the pathways of lipid uptake, distribution, storage, and use in the cell (19, 20, 21). Proteomic profiling of liver and adipose LDs revealed that these droplets contain specific structural proteins and enzymes from multiple metabolic pathways (12, 22). These findings implicate LDs in the regulation of hepatic and adipocyte metabolic processes.

Lipids are the primary fuel utilized for cardiac energy production. Normally about 60% to 80% of the total amount of ATP used by the heart is generated by mitochondrial oxidation of lipids (23, 24). Therefore, lipids have a direct role in heart function. In a normal heart, lipid uptake and oxidation are well balanced, and few droplets accumulate. Under pathological conditions such as in high fat diet–fed and genetically modified mice, the imbalance between cardiac lipid uptake and oxidation leads to accumulation of LDs as well as toxic lipid subspecies—such as diacylglycerols (DAGs), ceramides and acylcarnitines—and causes lipotoxic cardiomyopathy (25, 26, 27, 28). Heart LDs also accumulate during fasting but do not appear to hinder heart function (29). Similarly, LDs due to overexpression of the final enzyme in TAG synthesis, diacylglycerol acyl transferase (DGAT)1, doubles heart TAG content, but does not cause cardiac dysfunction (30) and improves response to ischemia (31). Thus, some but not all LDs are associated with pathology. While TAG is the most abundant constituent of the LD and heart TAG levels increase with obesity and the metabolic syndrome, TAG itself likely is not toxic (32).

We created one model of lipotoxic cardiomyopathy due to increased lipid uptake, oxidation, and accumulation by cardiomyocyte-specific overexpression of peroxisomal proliferator-activated receptor γ1 (MHC- *Pparg1*) (28). Even in this model, LDs alone did not appear to cause toxicity, as crossing the MHC-*Pparg1* transgene onto the peroxisome proliferator activated-receptor α knock out (*Ppara*^-/-^) background-corrected toxicity without reducing heart TAG (33). *Ppar-α* is the primary regulator of fatty acid oxidation in the heart and thus might prevent the production of excess reactive oxygen species by improving mitochondrial efficiency and promoting the oxidation of fatty acids (FAs) (34, 35, 36). PPAR-α also modulates the production of the TAG lipolysis enzymes hormone-sensitive lipase (HSL) and adipose TAG lipase (ATGL) (37) and its deficiency might negate the excess LD lipolysis and production of toxic lipids in the MHC-*Pparg* mice.

Transgenic expression of FA transport protein 1 (Fatp1), long-chain acyl-CoA synthetase 1 (Acsl1), Ppara, Pparg, and Perilipin5 (PLIN5) (38, 39) all lead to increased heart content of DAGs, ceramides, or both (26, 27, 28, 40). Moreover, failing human hearts also accumulate these toxic lipids (41). These data support the hypothesis that increased cellular levels of toxic lipids and not LDs per se promote heart dysfunction.

In this report, we characterized the LDs from mice with and without lipid toxicity. Specifically, we examined the lipidome and proteome of LDs and heart tissues in MHC-*Pparg1* and MHC-*Dgat1* mice. No previous studies have specifically assessed the composition of mouse heart LDs that accumulate in physiological and pathological states. Moreover, we assessed whether the accumulated heart DAGs and ceramides were a component of the LDs.

## Materials and methods

### Animal models

MHC-*Pparg1* (28), MHC-*Dgat1* (30), and C57BL6/J mice (Jackson lab) were used in this study. Male mice were used for a majority of experiments to avoid introducing variability due to hormonal changes in female mice and because the cardiac phenotypes were most pronounced in males. 12-14-week-old MHC-*Pparg1* and MHC-*Dgat1* mice and their littermates were fed a rodent chow diet (Cat No 5053, LabDiet). The day before the experiment, mice were fasted overnight (16 h). All mice were housed in a facility with controlled conditions (25°C) and a 12-h light-dark cycle (light from 7:00 am to 7:00 pm) until euthanasia by isoflurane. The experimental protocols were approved by the Institutional Animal Care and Use Committees, IACUC, at New York University Grossman School of Medicine.

### LD isolation

The LDs were isolated using a modification of established techniques (42, 43, 44, 45, 46). Briefly, after perfusion with 10 ml of PBS, the heart was removed and dissected free of fascia and connective tissues. Depending on the experimental needs, 5 hearts from the same genotype were pooled into one sample and then cut into small pieces and resuspended in 10 ml of homogenization buffer containing 250 mM sucrose and 20 mM tricine (pH 7.8) with cOmplete™ protease inhibitors cocktail (catalog 11836170001, Roche) on ice. A tight-fitting Dounce homogenizer was used to homogenize the pooled heart fragments on ice until the tissue was completely homogenized. The homogenate was centrifuged at 300g for 10 min at 4°C to remove nuclei, cell debris and unbroken cells. The supernatant was transferred into a SW40 tube, and an additional 2 ml of LD floating buffer was added (20 mM HEPES, 100 mM KCl, 2 mM MgCl2, pH 7.4) on the upper layer, and centrifuged at 10,000g for 1 h at 4°C (SW-41Ti rotor, Beckman Coulter) to separate the LDs from other cellular fractions. The LD fraction at the top of the tube was then transferred to an Eppendorf tube. After reloading 2 ml of LD floating buffer and centrifuging at 30,000g for 1 h at 4°C, the LD layer was collected and transferred to an Eppendorf tube containing the first collected LDs. Then the collected LDs were washed two additional times for 5 min each to remove loosely bound and/or contaminating proteins. To separate the membrane proteins and lipids of LD samples, 1 ml of cold acetone was added to the LD fraction. After incubating overnight at −20°C and centrifuging at 10,000 g for 5 min at 4°C, the organic phase was collected for lipidomics analysis, and the pellet was air dried and dissolved in 20 μl of RIPA (Abcam, Cat No: ab15603) buffer for proteomics analysis.

### LD staining

Cardiac ventricular tissues from 16-hour–fasted 4-month-old male mice were embedded in Tissue-Tek OCT Compound (Sakura). Frozen sections (10-μm) were fixed in 10% formalin for 30 min and then washed 3 times in PBS. The fixed sections were then incubated with 4 μM BODIPY 493/503 dye (catalog D3922, Invitrogen, Thermo Fisher Scientific) for 30 min at room temperature. After washing three times, sections were stained with ProLong Gold Antifade Mountant with DAPI (Thermo Fisher Scientific) and covered with glass coverslips (VWR, Avantor). The digital images were obtained with a Leica confocal microscope.

Co-staining of LDs was performed with the neutral lipid dye BODIPY 493/503 and the mitochondrial dye MitoTracker deep red (MitoTracker,Cat No. M22426, Invitrogen). In short, 1 μl of re-suspended LDs was combined with 1 μl PBS buffer supplemented with 1 μM MitoTracker deep red and 1 μM BODIPY 493/503 on a 1.0 mm glass slide (EMS 71867) and covered with #1.5 thickness cover glass (EMS 72222). Imaging was performed using 63 X Apochromat oil-immersion lens.

### Transmission electronic microscopy (TEM)

Left ventricles from 16-hour–fasted 4-month-old male mice were fixed with 2.5% glutaraldehyde and 2% paraformaldehyde in 0.1 M sodium cacodylate buffer (pH 7.2) for 2 h and then postfixed with 1% osmium tetroxide (1.5 h at room temperature), processed, and embedded in EMbed 812 (Electron Microscopy Sciences). Ultrathin sections (60-nm) were cut (Leica UC6 microtome), mounted onto 200 mesh copper grids, and stained with uranyl acetate and lead citrate. Stained grids were examined with a Philips CM-12 electron microscope and photographed with a Gatan (4 k × 2.7 k) digital camera.

### Immunofluorescence staining

Cardiac ventricular tissues from 4-month-old male mice were embedded in Tissue-Tek OCT compound (Sakura) and cryosectioned at 6 μm. Sections were fixed in 10% formalin for 30 min, washed three times with PBS, and blocked in 5% BSA/PBS for 1 h at room temperature. Primary antibodies against HSL (Cell Signaling Technology, 4107S), LC3B (Thermo Fisher, #81004-1), or PLIN5 (Proteintech, #26951-1-AP) were applied at 1:250 and incubated overnight at 4°C. After washing, sections were incubated with Alexa Fluor 488 donkey anti-rabbit IgG (Life Technologies, A21206; 1:500) for 1 h at room temperature. Slides were mounted with ProLong Gold Antifade Mountant containing DAPI (Thermo Fisher Scientific), and images were acquired using a KEYENCE microscope.

### LC-MS and protein quantification

Proteins were precipitated from either LDs or tissues. Samples were washed once with cold (−20°C) acetone. After the acetone was removed, remaining traces were eliminated in speed vac. Proteins were solubilized in buffer composed of 5% SDS, 20 mM CAA, 10 mM TCEP, and 50 mM TRIS pH7.4 (30 min at 90°C). Proteins were then purified by SP3 method. Briefly, proteins were precipitated on SP3 magnetic beads by adding EtOH (final 50%) with subsequent washes in EtOH. Enzymatic digestion was carried out in 25 mM TRIS pH7.4 with trypsin o/n at 37°C. Digestion was halted by acidification with fatty acid (FA) and peptides were loaded on Evosep C18 tips for Liquid Chromatography Mass Spectrometry (LC-MS/MS) analysis on HFX instrument operating in DIA mode. LC separation was done on EvosepOne system using Whisper 20SPD LC method (58 min gradient).

Tissue samples for proteomic analysis were transferred to 96 well plates and diluted 10x in 5% SDS 50 mM Tris (pH7.4), 20 mM CAA and 10 mM TCEP. Proteins were solubilized, reduced and alkylated at 90°C for 30 min. Proteins were then precipitated on SP3 beads utilizing the same procedure as for the LD (see previous). Proteins were enzymatically digested into peptides with trypsin at 37°C in 25 mM TRIS, pH 7.4. 5% of each digest was loaded onto Evosep tips for MS analysis using the same setup as for LD samples.

Peptide digests were acidified with FA (to final 0.5%) and loaded on C18 Evosep tips for immediate LC-MS/MS analysis using Evosep One LC system (Whisper 20SPD method, 58 min LC gradient) coupled with Orbitrap HFX instrument operating in DIA acquisition mode. High-resolution full MS spectra were acquired with a resolution of 120,000, an AGC target of 3e6, with a maximum ion injection time of 60 ms, and scan range of 350–1,650 m/z. Following each full MS scan 22 data-independent HCD MS/MS scans were acquired at the resolution of 30,000, AGC target of 3e + 6, stepped NCE of 22.5, 25 and 27.5.

Data-independent acquisition (DIA) data were analyzed using Spectronaut software (https://biognosys.com/shop/spectronaut) and searched in direct DIA mode against the SwissProt subset of the Mouse uniprot database (http://www.uniprot.org/). Database search was performed in integrated search engine Pulsar. For searching, the enzyme specificity was set to trypsin with the maximum number of missed cleavages set to 2. Oxidation of methionine was searched as variable modification; carbamidomethylation of cysteines was searched as a fixed modification. The false discovery rate (FDR) for peptide, protein, and site identification was set to 1%. Protein quantification was performed on MS2 level using the 3 most intense fragment ions per precursor.

Protein imputation was performed using the DreamAI algoritm, as described (47). Differential protein expression between MHC-*Pparg1* and MHC-*Dgat1* mice and their corresponding WT controls was then completed using a two-sample unpaired *t* test with multiple testing correction adjustment using the Benjamini-Hochberg approach (48). The volcano plots were plotted using the EnhancedVolcano package in R and heatmaps were generated using the pheatmap package in R or Excel. Pathway analysis was completed using Ingenuity Pathway Analysis (IPA) software (QIAGEN) using the “core analysis” and “comparison analysis” functions were used to identify pathway enrichment and upstream regulators.

### Mass spectrometric and lipid quantification

Lipids for lipidomics analysis were extracted from tissues and LDs collected from a previous protein extraction method. Tissue samples were weighed to 23 mg wet weight and normalized to 1 ml of methanol spiked with Avanti Lipid standards. Tissue was homogenized, and LDs from protein extraction were in acetone. Lipids were extracted using a modified Bligh-Dyer method by taking 60 μl of sample in a 1:1:2 concentration of homogenate, LCMS grade water, spiked Avanti Standards and chloroform. Lipids from acetone were extracted using a modified Bligh-Dyer method by taking 60 μl of sample in a 1:1:2 concentration of LD solution, LCMS grade methanol with spiked deuterated standards (Avanti Splash Lipidomix, product 330707), and chloroform. Specific internal lipid standards added in each run for family semiquantitation were: 15:0-18:1(d7) phosphatidylcholine (PC, 150.6 μg/ml); 15:0-18:1(d7) phosphatidylethanolamine (PE, 5.3 μg/ml); 15:0-18:1(d7) phosphatidylserine (Na Salt, PS, 3.9 μg/ml); , 15:0-18:1(d7) phosphatidylglycerol (Na Salt, PG, 26.7 μg/ml), 15:0-18:1(d7) phosphatidylinositol (NH4 Salt, PI, 8.5 μg/ml), 15:0-18:1(d7) phosphatidic acid (Na Salt, PA, 6.9 μg/ml), 18:1(d7) lysophosphatidylcholine (LPC, 23.8 μg/ml), 18:1(d7) lysophosphatidylethanolamine (LPE, 4.9 μg/ml), 18:1(d7) cholesterol ester (CE, 329.1 μg/ml), 18:1(d7) monoacylglycerol (MAG, 1.8 μg/ml), 15:0-18:1(d7) diacylglycerol (DAG, 8.8 μg/ml), 15:0-18:1(d7)-15:0 triacylglycerol (TAG, 52.8 μg/ml), d18:1-18:1(d9) sphingomyelin (SM, 29.6 μg/ml and (d7) cholesterol (98.4 μg/ml). Both homogenized solution and LD solutions were vortexed, then centrifuged to form a separation of the top polar and a bottom nonpolar layer. The bottom layer containing chloroform was removed by vacuum centrifuge at 45°C for 30 min and resuspended in a 4:3:1 solution of LCMS grade Isopropanol, acetonitrile, and LCMS grade water. Both top and bottom layers were analyzed. Acquisition of sample data was performed using a 1,290 Infinity II liquid chromatography system coupled to an Agilent 6230B time of flight with electrospray ionization (Agilent). Mobile phases used in chromatography were as follows: Mobile phase A is a 5:1:4 solution of isopropanol, methanol, and water with 5 mM buffer of ammonium acetate and 0.1% of acetic acid. Mobile phase B is a 99:1 solution of isopropanol and water, with 5 mM buffer of ammonium acetate and 0.1% acetic acid. The chromatography gradients were as follows: percent mobile phase B was 0 at the beginning of the run, then increases to 20% after 5 min, 30% after 25 min, 95% after 35 min, and 0% after 38 min. Samples passed through a Zorbax C18 column with dimensions 4.6 × 50 mm with a 3.5-micron inner diameter (Agilent, Santa Clara CA). Column temperature was 50°C. Samples eluting were ionized via electrospray ionization under 300°C nitrogen gas temperature with a flow rate of 11 L/minute. Capillary voltage was 3,500 V. Acquisition of samples was performed in both positive and negative modes with separate injections.

Mass spectra were identified using Agilent MassHunter software (Agilent), and analysis was performed using Agilent Mass Profiler Professional (Agilent). The lipidomics result was normalized with Rab18 LDAMP, which localized directly in the monolayer surface of LDs. Additional analysis was performed using Python, Microsoft Excel, and GraphPad PRISM. All data shown in graphs are the mean ± SD. Statistical difference was determined using paired *t* test or one-way analysis of variance (ANOVA) multiple comparisons test, as specified in Fig. legends [no significant (ns), ∗*P* < 0.05, ∗∗*P* < 0.01, ∗∗∗*P* < 0.001].

## Results

### Isolation and purification of LDs from mouse heart by differential centrifugation

**Fig. 1:**
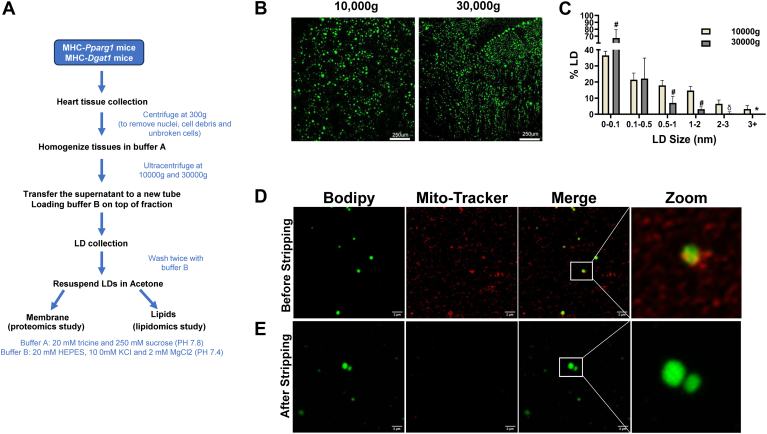
Development of a method for isolation of cardiac LDs. A: Outline of the workflow: Schematic of the experimental protocol used to isolate heart LDs. B: BODIPY staining showed wild type cardiac LDs centrifugated at different speeds and (C) Individual LD size was quantified using Fiji, and statistical analysis and figures were created with GraphPad software. D and E: LDs were co-stained by the neutral BODIPY 493/503 fluorescent dye and MitoTracker dye. Super-resolution confocal images of the LDs before (D) and after (E) stripping. Zoom indicates the physical amplification of insert. Data shown as means ± SD. ∗*P* < 0.05, #*P* < 0.01 and ^§^*P* < 0.001 compared with controls.

LDs float on top of aqueous buffers after centrifugation. All published methods for isolating LD from cells and tissues rely on differential centrifugation based on this feature of lipids (42, 44, 47, 49). The speed of centrifugation mainly depends on the size of the LDs. Compared with liver and adipose tissues, cardiac LDs are relatively small, low in abundance and always close to mitochondria, which makes isolation of cardiac LDs more challenging than other tissues. Considering these peculiarities, we designed the following experimental protocol in this study: (1) pooled five mice hearts to increase the amounts of LDs, (2) used low-speed (10,000g) and high-speed (30,000g) centrifugations to obtain large and small LDs, respectively, and avoid the breakage of large LDs caused by high speeds and loss of small LDs caused by low speeds, (3) washed twice after LD extraction to minimize the contamination of mitochondria and other organelles. The schematic diagram of LD extraction is shown in Figure 1A. To test the experimental protocol, we isolated LDs from C57BL/6J mice hearts to check the LD quality; WT mice develop LDs after overnight fasting (29). Staining the LDs with the neutral lipid dye BODIPY 493/503 showed that the LDs obtained by two different centrifugal speeds differ in size, the low-speed (10,000g) centrifugation yielded large LDs, and high-speed (30,000g) centrifugation yielded small LDs (Fig. 1B, C).

We then examined the purity of the LDs. We co-stained the LDs with the neutral lipid dye BODIPY 493/503 and the mitochondria dye MitoTracker deep red. Before washing, super-resolution confocal microscopy revealed that the LDs were surrounded by MitoTracker stained structures, indicating that mitochondria were preserved in the LDs (Fig. 1D). After washing twice, most of the LDs (95%) no longer had MitoTracker fluorescence (Fig. 1E). This result suggested that mitochondria were effectively removed from LDs after washing.

### Increased accumulation of cardiomyocyte LDs in MHC-Pparg1 and MHC-Dgat1

The role of LDs in cardiomyopathy remains unclear. Accumulation of intra-myocellular lipids in the heart is a commonly described feature of most animal models of obesity (50, 51). The importance of these LDs in cardiac function is unclear as they accumulate much sooner than any measurable functional defect. Within 2 weeks high fat diet leads to cardiac insulin resistance (52), thus the accumulated lipids alter heart metabolism. In contrast, the excess lipids in cardiomyocytes were associated with systolic dysfunction and rhythm disturbances in MHC-*Pparg1* mice (28, 53). MHC-*Dgat1* mice have increased cardiac lipid accumulation but normal heart function (30).

**Fig. 2:**
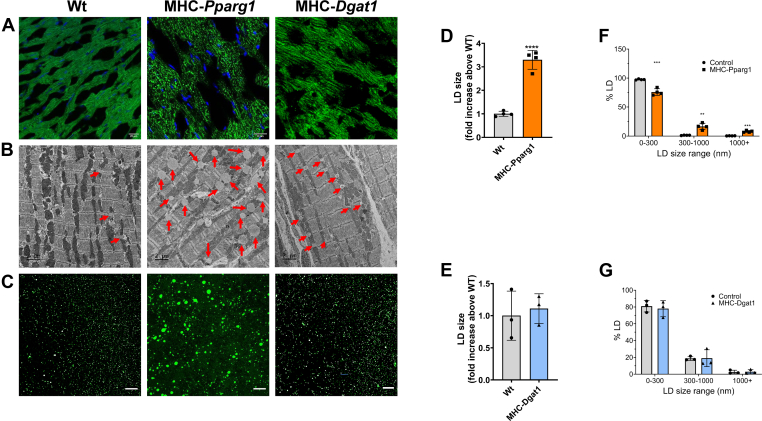
LDs from MHC-*Pparg1* and MHC-*Dgat1* hearts differ in size. A: Heart tissue sections from WT, MHC-*Pparg1* and MHC-*Dgat1* were stained with BODIPY 493/503 fluorescent dye. Scale bars: 50 μm. B: Electron micrographs (original magnification ×3,400) of heart tissues showing a significant increase in LDs within the sarcoplasm of cardiomyocytes from MHC-*Pparg1* and MHC-*Dgat1* mice. Red arrows indicate LDs. C: BODIPY 493/503 fluorescent dye stained the LDs isolated from the MHC-*Pparg1* and MHC-*Dgat1* mouse hearts. Scale bars: 50 μm. Average LD size (D and E) and LD size ranges (F and G) in MHC-*Pparg1* (n = 4) and MHC-Dgat1 (n = 3) and their littermate controls (n = 3 for each group). . Individual LD size was quantified using Fiji, and statistical analysis and figures were created with GraphPad software. Data shown as means ± SD. ∗∗∗∗*P* < 0.001 compared with WT controls.

We first stained cardiac ventricular sections with BODIPY 493/503 to examine cardiac LDs in these mice. Fluorescence microscopy revealed that the number of LDs in the hearts of MHC-*Pparg1* and MHC-*Dgat1* mice were increased compared with fasted WT controls (Fig. 2A). As assessed by TEM, individual LD size in controls was approximately 0.1–0.5 μm, whereas in MHC-Pparg1 mice LDs were 0.3–1.9 μm. Although MHC-*Dgat1* mice had more LDs, LD size did not change compared with overnight fasted non-genetically altered littermate controls and were smaller than those of MHC-*Pparg1* mice (Fig. 2B).

### Lipolysis and autophagy correlate with LD size

We next isolated the LDs from the hearts of WT controls (n = 3), MHC-*Pparg1* (n = 4) and MHC-*Dgat1* (n = 3) mice. The analysis was of samples obtained using 5 mice hearts; the data are therefore derived from 15-20 animals. We used BODIPY 493/503 to stain isolated LDs as described above, we confirmed that the LDs from MHC-*Pparg1* mice were significantly larger than WT controls and MHC-*Dgat1* mice. In MHC-*Pparg1* mice, the size of LDs was increased about 3.5-fold compared to those in controls, and the size of LDs in MHC-*Dgat1* mice was not significantly different from those in the fasted control mice (Fig. 2C–E). We compared the LD size range between MHC-*Pparg1* and MHC-*Dgat1* with their corresponding littermate controls. In the MHC-*Pparg1* LDs, the proportion of small LDs (0–300 nm) significantly decreased, while the proportion of medium (300–1,000 nm) and large (>1,000 nm) LDs increased. The LD sizes in MHC-*Dgat1* were not significantly different from those in their littermate controls.

**Fig. 3:**
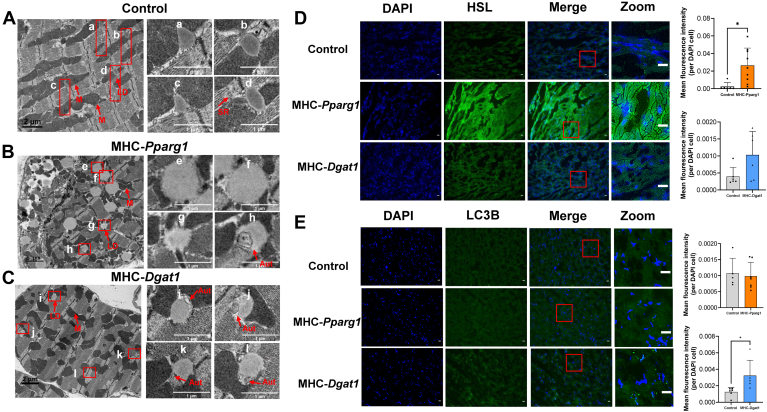
LDs from MHC-*Pparg1* and MHC-*Dgat1* hearts appear to use different metabolic routes. Left panels: electron micrographs of heart tissue sections from (A) WT control, (B) MHC-*Pparg1* and (C) MHC-*Dgat1* mice. Right panel: the area highlighted by red boxes in the left panel were enlarged. a-d: WT cardiomyocyte LDs in the cytoplasm are partially surrounded by mitochondria, and (a, c and d) are in close contact with the SR. e-h: MHC-Pparg1 cardiomyocytes undergo lipolysis; (e-g) LDs were large in shape, with irregular and completely ruptured membranes, and (h) autophagosome was direct contact with lipolytic LD. i-l: LD are in close contact with autophagosome structures in MHC-*Dgat1* cardiomyocytes. D: Immunofluorescence staining of HSL and (E) LC3B antibodies in MHC-*Pparg1* and MHC-*Dgat1* heart tissues. Scale bars: 20 μm. The area highlighted by red boxes in the left panel were enlarged and the quantifications of mean fluorescence intensity per DAPI cell are shown on the right panel. Data shown as means ± SD. ∗*P* < 0.05. Abbreviations: AUT, autophagosome; M, mitochondria; SR, sarcoplasmic reticulum.

LD size might impact structure and function; thus, we used TEM to examine the ultrastructure and shape of the LD membrane. These images are shown in Figure 3A–C. The area highlighted in red boxes from the left panel were enlarged. While the sarcoplasmic reticulum is smooth tubular and has a single membrane-bound structure involved in calcium storage and release, the autophagosome is a round double membrane vesicle and initiates breakdown of the LDs for energy production (54). TEM showed that in WT and also littermate control cardiomyocytes (Fig. 3A), LDs were located near mitochondria (a-d) and sarcoplasmic reticulum (a, c and d). In MHC-*Pparg1* cardiomyocytes (Fig. 3B), LDs exhibited large irregular shapes and outlines (e and f) and the LD surface was completely ruptured (g) and autophagosomes (h) directly contacted these LDs that were undergoing lipolysis. In contrast, MHC-*Dgat1* cardiomyocyte LDs (Fig. 3C) were similar in size to controls and were fused with autophagosome structures (i-l). To further verify whether lipolysis and autophagosome were involved in the LD metabolic pathway in these mice, we stained the hearts of these two groups of mice with immunofluorescence antibodies against HSL and LC3B, which are markers of lipolysis and autophagosome formation, respectively. The fluorescence intensity of HSL in MHC-*Pparg1* hearts was significantly greater than that in control and MHC-*Dgat1* hearts, while the intensity of LC3B in MHC-*Dgat1* hearts was greater than in control and MHC-*Pparg1* hearts (Fig. 3D, E). The quantifications of mean fluorescence intensity per DAPI cell are shown on the right panel of each group. Thus, these results collectively indicated that the intracellular metabolic processes affecting LDs were different in these two genotypes.

### Proteomic analysis of LDAMPs in MHC-*Pparg1* and MHC-*Dgat1* mice

**Fig. 4:**
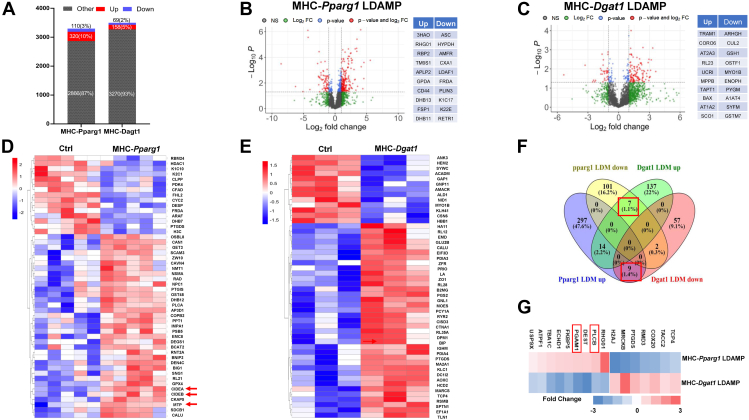
LDAMPs differ between pathologic MHC-*Pparg1* and physiological MHC-*Dgat1* mice (A and B). A: Bar graphs summarize differentials in the proteome of LDM in MHC-*Pparg1* and MHC-*Dgat1* mice. B and C: The volcano plot representing significantly differentially abundant (*P*-value <0.05) proteins in MHC-*Pparg1* and MHC-*Dgat1* LDMs and their respective controls. Red region reflects proteins increased or decreased by a ratio of >1.3 with *P* < 0.05. The top 10 up and down proteins are listed in the right table. D and E: Hierarchical clustering of differentially abundant top fifty proteins representing normalized abundance values in MHC-*Pparg1* and MHC-*Dgat1* LDMs. F: Venn diagram showing the count of overlapped and specific proteins (*P* < 0.05) that were significantly increased and decreased in MHC-*Pparg1* and MHC-*Dgat1* LDMs. The number of proteins that are common or unique is expressed along with the corresponding percentage relative to the total number of differentials expressed proteins. The red frame shows overlapping proteins with opposite expression. G: The heat map shows overlapping proteins that were inconsistently expressed in MHC-*Pparg1* and MHC-*Dgat1* LDM.

Proteomic studies of LDs in mice have been reported for liver (47, 55), skeletal muscle (45) and adipose tissue (56, 57). A heart proteomic study was reported, but from rat hearts (58). To characterize the LDAMPs in mice with and without lipid toxicity, we isolated LDs from the hearts of MHC-*Pparg1* and MHC-*Dgat1* mice according to the method described above. Then we performed LC-MS based proteomic analyses to identify and quantify proteins in each group of LDs. The proteome profiles in this study are summarized in Figure 4A. We identified 3296 LDAMPs in the MHC-*Pparg1* model and 3497 LDAMPs in the MHC-*Dgat1* model. The number of LDAMPs identified by our proteome analysis are similar to those reported by Bosch *et al.* (49). Compared to the LDMs from control fasting mice, 13% of LDAMPs from MHC-*Pparg1* and 7% of proteins MHC-*Dgat1* differed. Thus, 430 differential LDAMPs were detected in MHC-*Pparg1* mice, among those, 320 proteins (10% of total) were increased, and 110 proteins (3% of total) were decreased. The number of differential proteins in MHC-*Dgat1* LDM was less than that in MHC-*Pparg1* LDM. There were 227 differential LDAMPs, accounting for 7% of total detected proteins, of which 158 (5%) proteins were increased, and 69 (2.0%) proteins were decreased. The difference in the differential protein ratio in MHC-*Pparg1* LDM and MHC-*Dgat1* LDM may reflect the effect of these two transgenes on cardiomyocytes and resulting pathological changes in the LDAMPs.

Differential protein analysis on the LDAMP proteomes from MHC-*Pparg1* and MHC-*Dgat1* mice compared with WT showed important changes between models. In the MHC-*Pparg1* comparison, a total of 430 proteins were different (320 up and 110 down, *P* < 0.05) (Fig. 4B). In the MHC-*Dgat1* comparison, 227 proteins (158 up and 69 down) were found at the same significance cutoff (Fig. 4C). The top 10 up- and downregulated proteins (*P* < 0.05) are also listed in the tables to the right of the volcano plot (Fig. 4B, C), which include proteins involved in formation of glycerol-3-phosphate, lipid biosynthesis, and phospholipid peroxidation.

Protein expression data from the top 50 differential LDAMPs (*P* < 0.05) in the MHC-*Pparg1* and MHC-*Dgat1* LDs are shown in Figure 4D, E. Among the top 50 differential proteins in MHC-*Pparg1* LDAMP, 35 proteins were increased and 15 were decreased. Gene interaction analysis of correlated proteins revealed that several functionally connected protein networks, such as CIDEA, CIDEB, and MTP that are commonly recognized as LD size related proteins (59), were upregulated. This result is consistent with our finding of large LDM disruption by transmission EM in MHC-*Pparg1* cardiomyocytes. The clustering results showed that some of the major proteins involved in ER-to-Golgi traffic (AP3D1, COPB2 and EMC8), phospholipid hydroperoxides (GPX4), phagolysosome and lysosome degradation (PLCA and PPT1), exocytosis regulator (SNG1), sphingolipid and peroxisome lipid metabolism (DEGS1 and GET3), and fatty acyl-CoA cholesterol biosynthesis (DHB12), were accordingly increased in MHC-*Pparg1* LDAMPs.

To explore the molecular functions of proteins between the *MHC-Pparg1* and MHC-*Dgat1* LDAMPs, we used Venn diagram to compare the composition of significantly increased- and decreased proteins (*P* < 0.05). A total of 14 differential up-regulated and 2 down-regulated proteins overlapped between the MHC-*Pparg1* and MHC-*Dgat1* LDAMP (Fig. 4F and Supplemental Table S1). Enriched KEGG pathway analysis showed that these 14 proteins (Supplemental Table S2), commonly increased in both groups, were involved in synaptic vesical cycle, endocytosis and fatty acid biosynthesis and degradation pathways (FDR < 5%). Of the 16 dysregulated proteins in MHC-*Pparg1* LDAMP, 9 were upregulated and 7 were down-regulated. Interestingly, expression patterns of these proteins were opposite in MHC-*Dgat1* LDAMP (Fig. 4G). Phosphoinositide-specific phospholipase C (PLC) and phosphoglycerate mutase 2 (PGAM), important enzymes for the DAG metabolism (60) and glycolysis pathways (61), respectively, were increased in MHC-*Pparg1* but decreased in MHC-*Dgat1* LDAMPs.

**Fig. 5:**
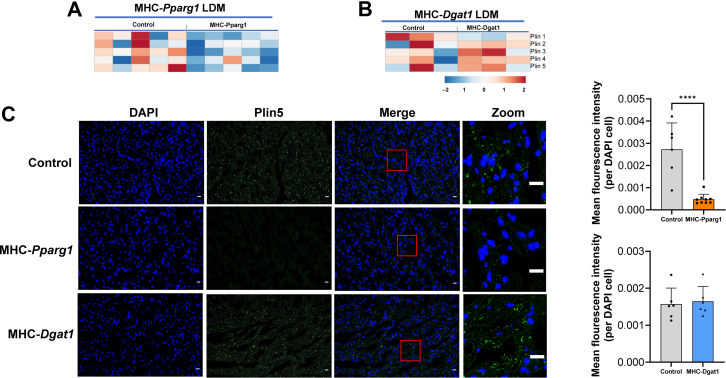
The reduction of PLIN protein expression in MHC-*Pparg1*. A and B: The heat map showing perilipin proteins in the MHC-*Pparg1* and MHC-*Dgat1* mice. Red denotes higher than average expression, blue denotes lower than average expression. C: Immunofluorescence staining of PLIN5 antibody in MHC-*Pparg1* and MHC-*Dgat1* heart tissues. Scale bars: 20 μm. Right panel: the area highlighted by red boxes in the left panel were enlarged.

PLIN family proteins regulate LD stability and lipid turnover, consequently controlling overall lipid metabolism (62). PLIN proteins have tissue-specific expression patterns, and PLIN3 and PLIN5 are highly expressed in cardiac muscle (63). As mentioned above, the size of the LD in MHC-*Pparg1* mouse heart was approximately 3.5-fold larger than that in MHC-*Dgat1* hearts. To study whether the size of LDs was associated with different or more PLINs, we illustrated enrichment of PLIN proteins by a heat map (Fig. 5AF, B). We initially expected that MHC-*Pparg1* LDAMP with large LDs would have abundant PLIN proteins. Surprisingly, all PLIN proteins in these large LD (MHC-*Pparg1*) were reduced compared with the controls, especially PLIN3 and PLIN5 (Fig. 5A). In MHC-*Dgat1* LDAMPs, except for a slight decrease in PLIN1, Plin2 to PLIN5 ratio was not different than the control group (Fig. 5B). This discrepancy between larger LD with fewer membrane proteins suggested that increased lipid core leads to a proportionately greater amount of surface phospholipid monolayer and less need for PLIN mediated membrane stabilization. Alternatively, the surface of the MHC-*Pparg1* LDs had proportionately less PLINS due to the association of other proteins.

PLIN5 is the only PLIN to directly bind to ATGL and protect against TAG hydrolysis by ATGL (64). We used immunofluorescence staining to assess cardiac PLIN5 protein expression in these mice. As shown in Figure 5C, the amount of PLIN5 protein in the MHC-*Pparg1* mice heart was significantly reduced compared with the controls, whereas PLIN5 in the heart of MHC-*Dgat1* mice was not different from that in the controls. The reduction of PLIN5 in MHC-*Pparg1* mice indicated a loss of LD membrane stability and increased LD lipolysis in these mice.

**Fig. 6:**
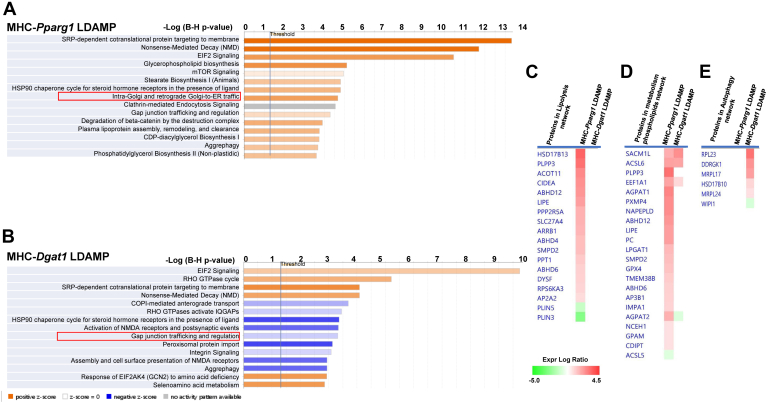
IPA analysis of proteome enrichment pathways is consistent with differences in LD metabolism. A and B: Canonical pathways of LDM proteins determined by IPA. Positive Z-score indicates increased and negative Z-score indicates decreased proteins, shown in orange and blue bars (shades), respectively. C-E: Heat map showed comparison of proteins involved in lipolysis (C), autophagy (D), and metabolism phospholipids pathway (E) in MHC-*Pparg1* and MHC-*Dgat1* LDM.

We then identified canonical pathways enriching the significantly dysregulated proteins in MHC-Pparg1 LDAMPs and MHC-*Dgat1* LDAMPs (FDR < 5%). A total of 112 and 42 enriched canonical pathways were identified in MHC-*Pparg1* LDAMPs and MHC-*Dgat1* LDAMPs, respectively. Among them, 80 (71.4%) were activated, 7 (6.2%) were inhibited and 25 (22.3%) were not activated in MHC-*Pparg1* LDAMPs, and 17 (40.5%) were activated and 18 (42.8%) were inhibited and 7 (16.7%) were not activated in MHC-*Dgat1* LDAMPs. The top 15 representative pathways ranked according to −log (FDR) are shown in Figures 6A, B.

IPA graphical summary showed that lipolysis, metabolism of phospholipids and synthesis of lipids were predicted to be activated in MHC-*Pparg1* LDAMP (Supplemental Fig. S1A). Consistent with these results, comparative analysis exhibited that lipolysis and metabolism of phospholipid related proteins, such as AGPAT, LGPAT, CIDEA and LIPE, were greater in MHC-*Pparg1* LDAMPs compared with MHC-*Dgat1* LDAMPs (Fig. 6C, D). The comparative canonical pathway analysis revealed that the ceramide and sphingolipid metabolism signaling pathways were activated in MHC-*Pparg1* compared with MHC-*Dgat1* LDAMPs (Supplemental Fig. S2A–F). A comparative analysis of ER autophagy proteins using IPA analysis showed that several proteins in ER autophagy network were activated in MHC-*Dgat1* LDAMPs but not in MHC-*Pparg1* LDAMPs (Fig. 6E). These data suggest that the ER autophagy pathway was upregulated in the MHC-*Dgat1* LDAMPs.

### Translational multi-omic evidence of reduced energy production

**Fig. 7:**
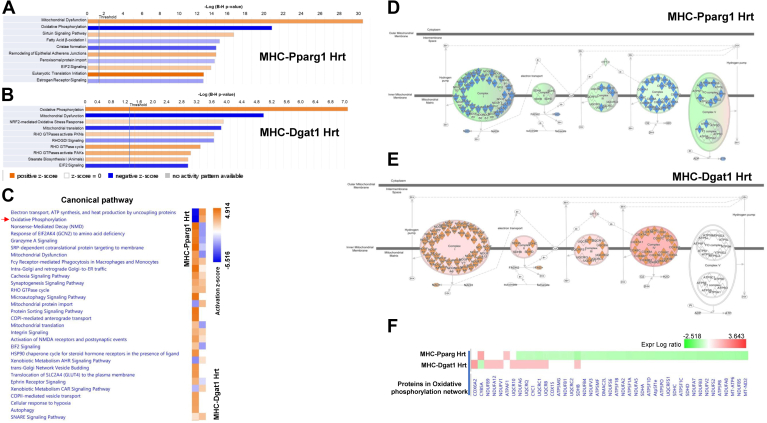
IPA analysis of heart tissue proteome of MHC-*Pparg1* and MHC-*Dgat1* mice suggests differences in oxidative phosphorylation. A and B: Canonical pathways of heart proteins. Positive Z-score indicates upregulated and negative Z-score indicates downregulated proteins, shown in orange and blue bars (shades), respectively. C: IPA comparison analysis of significant heart proteome enrichment pathways in MHC-*Pparg1* and MHC-*Dgat1* hearts. D and E: The schematic diagram presented the individual proteins involved in oxidation phosphorylation pathway in MHC-*Pparg1* and MHC-*Dgat1* LD. F: Heat map showed comparison of proteins involved in oxidative phosphorylation pathway.

A hallmark of heart failure with reduced ejection fraction is a decrease in heart energy production. To assess whether changes in protein levels MHC-*Pparg1* and MHC-*Dgat1* hearts reflected future changes in heart function, we performed a canonical pathway analysis of heart proteins. The top 10 enriched pathways are shown in Figure 7. In the MHC-*Pparg1* hearts (Fig. 7A), the most significantly enriched pathways were activation of mitochondria dysfunction, inhibition of oxidative phosphorylation and FFA oxidation. These results suggested that mitochondrial oxidative phosphorylation pathways were impaired in the hearts of these mice. In contrast to MHC-*Pparg1*, the canonical pathway analysis of MHC-*Dgat1* mouse heart revealed activated pathways of oxidative phosphorylation as well as inhibition of mitochondrial dysfunction (Fig. 7B and Supplemental Fig. 3A). We then compared the canonical pathways using IPA, oxidative phosphorylation was inhibited in the MHC-Pparg1 heart but was activated in the MHC-*Dgat1* heart (Fig. 7C and Supplemental Fig. 3B). The schematic diagram presented the individual proteins involved in oxidation phosphorylation pathway in these mice (Fig. 7D, E). The proteins involved in the oxidative phosphorylation network were shown by heat map (Fig. 7F). Together, these results suggested that differences in LD morphology and proportion correlated with alterations in the cardiac lipid metabolism pathways and mitochondrial oxidative phosphorylation.

### Lipidomic analysis

**Fig. 8:**
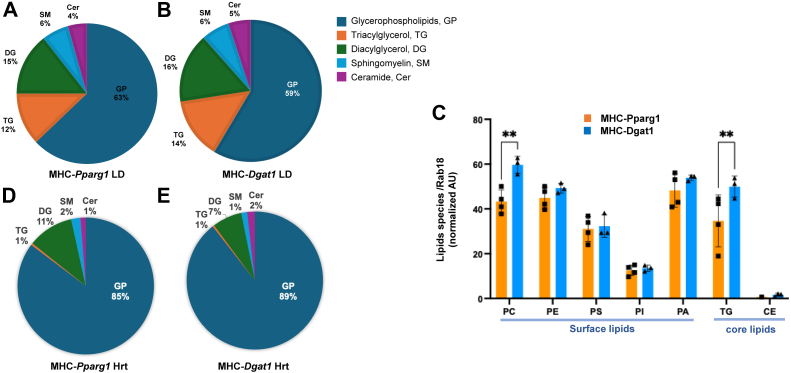
LD and heart lipid analysis. A and B: Proportion of lipids and (C) phospholipids in LDs isolated from 20 mice, divided into 4 groups. D and E: Whole heart lipid analysis, (n = 3). All data are shown as mean ± SD, ∗*P* < 0.05, ∗∗*P* < 0.01 in MHC-*Pparg1* compared to MHC-*Dgat1*.

To investigate whether the content of toxic core and surface lipids vary, and whether the demand for surface phospholipids is balanced with the core lipid volume, we next assessed the TAGs and phospholipids in LDs of MHC-*Pparg1* and MHC-*Dgat1* by targeted lipidomic analysis. The pie graph in Figure 8A, B showed the distribution of phospholipid, sphingolipids and core lipids in LDs of MHC-*Pparg1* and MHC-*Dgat1*. Compared with MHC-*Dgat1*, the proportions of toxic lipids—DAGs, SM and ceramides—were not increased in LDs of MHC-*Pparg1*. The percent core lipids, typically TAGs, decreased by 2%, and the monolayer phospholipid increased by 4% in MHC-*Pparg1* LDs. To extend these observations and determine the effects of differential lipid species on their proportions, we then compared the monolayer phospholipid and core lipid species in these two LDs (Fig. 8C). The composition of the phospholipid monolayer of LDs is mostly composed of phosphatidylcholine (PC), phosphatidylethanolamine (PE), and, to a lesser extent, phosphatidylinositol (PI) and phosphatidylserine (PS) (2, 62). We found that phosphatidylcholine (PC), which accounts for approximately 50%–60% of LD surface phospholipids in mammalian cells (2, 65) was decreased by approximately 27% (*P* < 0.01) compared with MHC-*Dgat1*. Thus, the decrease in PC content in MHC-*Pparg1* LDs correlated with the large size of these LDs. However, other phospholipids of the LD membrane, such as PE, PS, and PI, which contribute to LD stability, were unchanged in MHC-*Pparg1* compared to MHC-*Dgat1*. In part, we expect these lipidomic results to reflect a composite that includes the presence of large numbers of small LDs in all hearts. Moreover, it is possible that despite our attempts, phospholipids from other members remained attached to the isolated LDs.

We also measured the overall lipid content in the MHC-*Pparg1* and MHC-*Dgat1* hearts (Fig. 8D, E) to determine whether “toxic” lipids, which were not increased in the LDM, were increased in the MHC-*Pparg1* hearts. The amount of DAG, an intermediate product of lipid metabolism considered toxic, was greatest in the hearts of the MHC-*Pparg1* mice. Thus, it is likely that cytosolic and not LD species are pathologic.

## Discussion

There have been limited studies of the composition of LDs in the heart. Our data show that hearts with lipotoxicity have very different LDs than hearts from mice that have increased TAG but no effect or even a benefit from the stored lipids. These differences include changes in the size and protein composition of the LDs. LDs are often viewed as a reservoir for storage of excess lipids and are able to protect cells from toxic effects of lipid overload (32). However, they also correlate with the presence of heart dysfunction. Using transgenic mouse models, we created conditions with excess lipid storage but opposite effects on heart biology and obtained data that explain the relationship between LDs and cardiomyocyte function in MHC-*Pparg1* and MHC-*Dgat1* mice. Specifically, we observed the following: (1) Toxicity was associated with larger LDs. (2) These large LDs were more likely to be undergoing lipolysis. (3) As had been described in brown adipose (56), the LDAMP from MHC-*Pparg1* hearts was relatively depleted of PLIN proteins that may serve as a barrier to lipolysis. (4) In contrast, MHC-*Dgat1* hearts had more LDs undergoing lipophagy. (5) LDAMPs associated with toxicity had greater amounts of enzymes associated with the production of toxic lipids such as DAGs, although the proportion of these lipids in the isolated LDs was not increased. 6) The large LD size in MHC-*Pparg1* led to the expected decreased in the percent of LD PC content. 7) Enzymes involved in production of toxic lipids were enriched in the LDAMPs from MHC-*Pparg1* mice.

Cardiac LDs have specific characteristics that distinguish them from LDs in other tissues. LDs in cardiomyocytes are generally smaller and less abundant compared to those in adipose tissue and liver (19, 42, 47, 49), making it challenging to isolate sufficient quantities for study. Cardiac LDs and mitochondria are physically tethered to facilitate FA transfer for β-oxidation. This tight association makes it challenging to completely separate from each other. Heart tissue is also composed of dense fibrous tissue, which can complicate the homogenization process required for isolating LDs. Therefore, the isolation of LDs from mouse heart is generally more difficult compared to LD isolation from other tissues. Previous studies revealed interactions between LDs and early endosomes (66), mitochondria (67), endoplasmic reticulum (ER) (2, 3, 19), and peroxisomes (68). For this reason, we revised and optimized the protocols used for LD isolation from other tissues. This optimization allowed us to produce relatively pure LD preparations for protein and lipid analysis.

Nonetheless, proteins associated with LDs may be due to associated mitochondria as well as adhering cytosolic proteins. We attempted to reduce the mitochondria contribution using an extra “washing” step. In this study, we co-stained LDs with the neutral lipid dye BODIPY 493/503 and the mitochondria dye MitoTracker to check the purity of LDs. Although the staining of mitochondrial structures was negative (Fig. 2E), we still found abundant expression of mitochondrial proteins in both MHC-*Pparg1* and MHC-*Dgat1* LDM (Supplemental Table S3). Our data are consistent with Liu *et al.*, who proposed that the stable interaction between LDs and mitochondria is maintained by a rivet-like protein structure that prevents their separation during ultracentrifugation (69). We also found more proteins associated with DAG production in the MHC-*Pparg1* mice. These enzymes are thought to primarily be associated with ER, and their association with the LDM might reflect greater levels of the enzymes in the cardiomyocytes. This latter explanation is consistent with the RNA sequence analysis (28). We also compared our data to that describing LDs in rat heart (58) and found that only 9.5% and 10.2% of LDAMPs in MHC-*Pparg1* and MHC-*Dgat1*, respectively, overlapped with those in the rat heart LDs.

We were struck by how the differences in proteomics correlated with differences in how the LDs are metabolized. Specifically, the reduction in LDAMP PLINs appeared to allow greater lipolysis by intracellular lipases. In adipocytes, PLIN1 appears to assist with ATGL and HSL association with the LDM (70). Adipose tissue is, however, specialized not to use the stored lipids but to export them from the cells to provide energy to distant tissues. In contrast, both hearts and brown adipose tissue (BAT) are highly energetically demanding and use their stored lipids to generate ATP. In these tissues, PLINs may protect the LDs from unnecessary lipolysis, and the uncontrolled release of FFAs, which can lead to lipotoxicity and oxidative stress.

Excess accumulation of lipids in cardiomyocytes leads to lipotoxicity, but LDs can function as storage organelles to protect cells from accumulation of toxic lipid species. LDs in BAT are considered non-toxic because their FFAs are rapidly channeled into mitochondrial β-oxidation and thermogenesis rather than being stored as harmful intermediates (71, 72). Fasting has significant effects on cardiac metabolism, particularly regarding LDs in cardiomyocytes. During fasting, the heart switches to increased fatty acid oxidation to meet its high energy demands and to preserve glucose. As a result, there is a rise in the uptake of circulating FAs from the bloodstream, which are stored in cardiomyocyte LDs as TAGs (44). Despite the common association of more LDs with reduced heart function, the heart like the skeletal muscle can use LDs as a source of energy, similar to the “Athlete Paradox” that occurs with chronic exercise (21, 73). In skeletal muscle and heart this biology of greater lipid storage and increased insulin sensitivity was reproduced by overexpression of *Dgat1* (66). Our EM findings of greater images of lipophagy in the MHC-*Dgat1* hearts indicate that release of lipid by this mechanism allows oxidation of the stored TAGs without creation of toxic products. The exact mechanisms responsible for channeling lipolysis versus lipophagy released lipids remain to be explored.

In summary, comparisons of LDs from two models of heart lipid accumulation led to surprising insights that may shed light on both heart lipotoxicity and the physiological use of stored lipids. Our proteomic and EM analyses show the metabolism of these droplets. In contrast, lipid analysis was less revealing and implicates non-LD lipids as the culprit causing heart dysfunction due to excessive lipid accumulation. These are likely created by lipolysis; thus, the reduced toxicity of MHC-*Pparg1* crossed onto the Ppara knockout background might be explained by reduced creation of toxic lipids in this model. Our results suggest that heart LD toxicity can be prevented—not by only preventing LD release of lipids, as ATGL deficiency leads to massive lipid accumulation and heart failure—but by altering the pathways by which lipids leave the LD.

## Data availability

All data are contained within the article.

## Supplemental data

This article contains supplemental data.

## Conflict of Interest

The authors declare that they do not have any conflicts of interest with the content of this article.

## Supplementary data

Supplementary Figures

Supplymentary Tables
