# Supplementary material for: Cardiac lipid droplets differ under pathological and physiological conditions
Source: J Lipid Res. 2025 Oct 1;66(11):100920. doi: 10.1016/j.jlr.2025.100920 (PMC12617763; doi:10.1016/j.jlr.2025.100920)

# Supplementary Figure 1

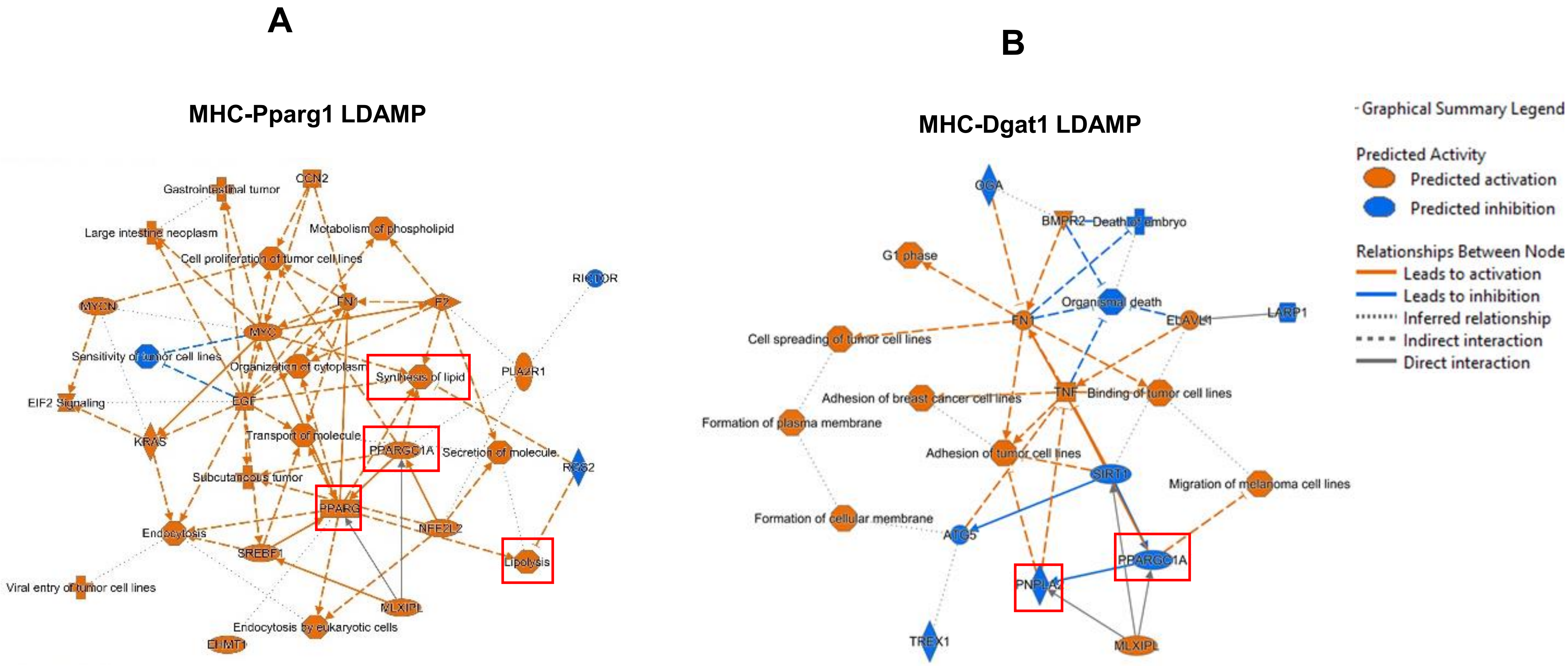

# Supplementary Figure 2

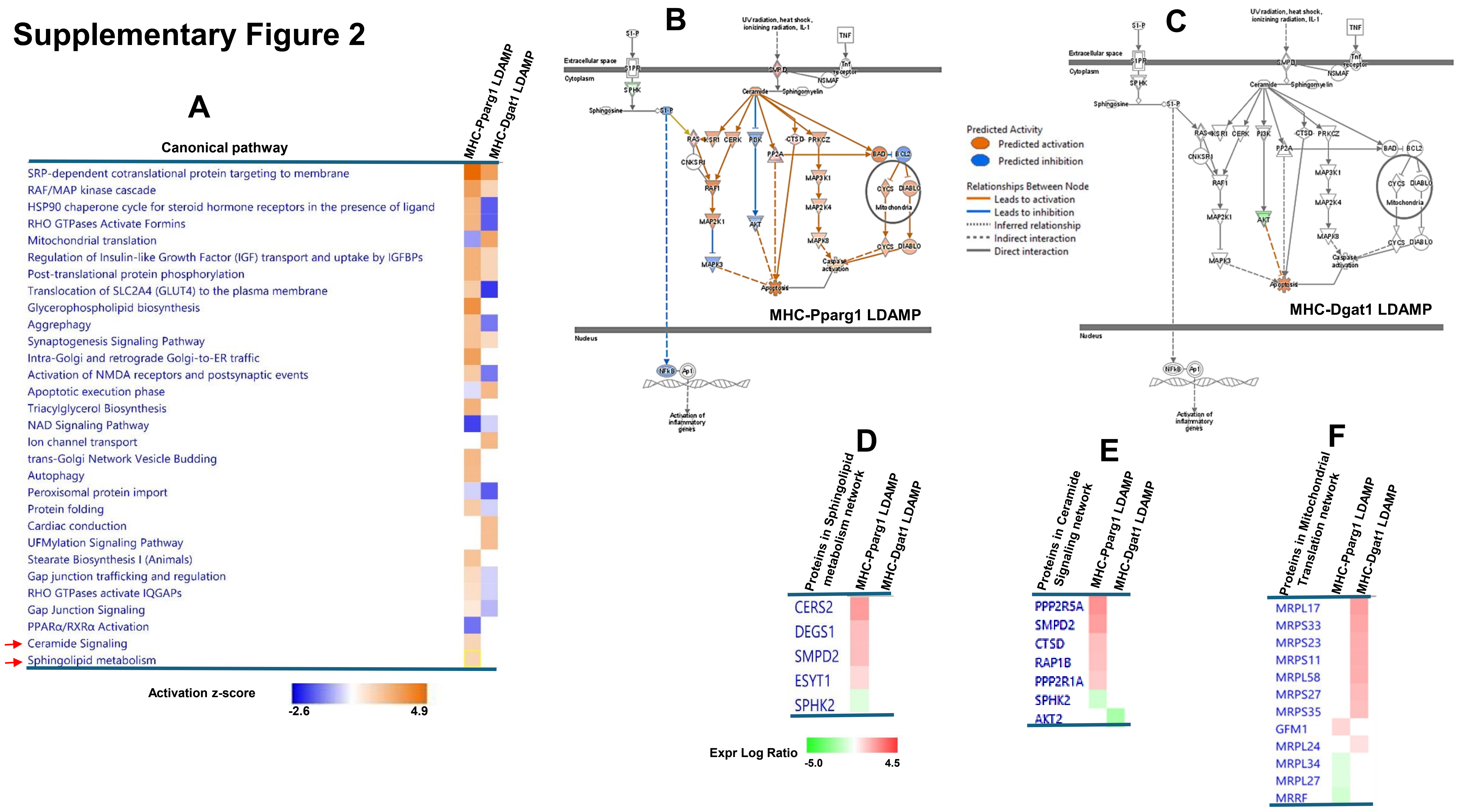

## Supplementary Figure 3

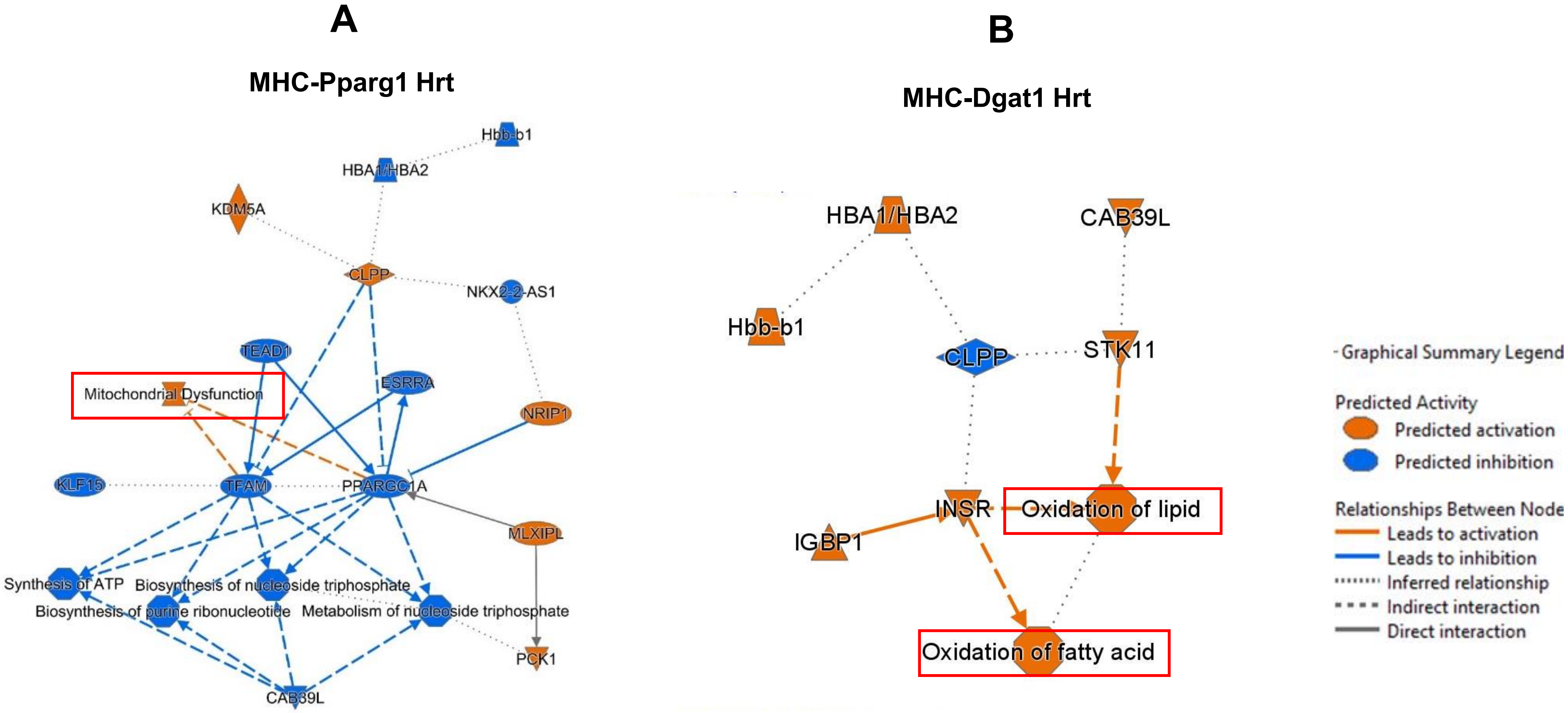

Supplement: Supplementary Figures [file mmc1.pdf]
