# Supplementary material for: Cardiac lipid droplets differ under pathological and physiological conditions
Source: J Lipid Res. 2025 Oct 1;66(11):100920. doi: 10.1016/j.jlr.2025.100920 (PMC12617763; doi:10.1016/j.jlr.2025.100920)
Supplement: Supplymentary Tables [file mmc2.pdf]

Supplementary Table 1. Proteins co-expressed in the MHC-Pparg1 and MHC-Dgat1 LDAMP

MHC-Pparg1 Up<br>MHC-Dgat1 Up	MHC-Pparg1 Up<br>MHC-Dgat1 Down	MHC-Pparg1 Down<br>MHC-Dgat1 up	MHC-Pparg1 Down<br>MHC-Dgat1 Down
ACSL6	RHG01	TCP4	GLUCM
EF1A1	PLCB	TACC2	GSTM7
SAC1	DEST	COX20	
PDIA4	PGAM1	RMD3	
CALU	FKBP5	PTGDS	
CAVN2	ECHD1	MRCKB	
TXD12	TBA1C	H2AJ	
AP2B1	ATPF1		
CSK21	USP9X		
SYFB			
AP2M1			
SYFA			
HS12A			
RS18			

Supplementary Table 2. KEGG pathway analysis of commonly increased proteins in MHC-Pparg1 LDAMP and MHC-Dgat1 LDM

Term	P-value	Adjusted P-value	Odds Ratio	Combined Score
Endocrine and other factor-regulated calcium reabsorption	0.001	0.009	65.1	481.8
Synaptic vesicle cycle	0.001	0.009	43.7	289.3
Fatty acid biosynthesis	0.013	0.046	90.4	395.7
Endocytosis	0.013	0.046	13.2	57.1
Huntington disease	0.019	0.053	10.8	42.9
Ferroptosis	0.028	0.059	38.4	136.7
Fatty acid degradation	0.030	0.059	36.5	128.5
Vibrio cholerae infection	0.034	0.060	31.3	105.4
Adipocytokine signaling pathway	0.047	0.065	22.5	68.8
PPAR signaling pathway	0.051	0.065	21.0	62.6
Thyroid hormone synthesis	0.051	0.065	20.7	61.5
Peroxisome	0.056	0.065	18.9	54.5
Protein processing in endoplasmic reticulum	0.113	0.122	9.0	19.5
Thermogenesis	0.151	0.151	6.6	12.4

Supplementary Table 3. Detection of Mitochondrial associated proteins in both MHC-Pparg1 and Dgat1 LDAMP

Genes	Names	UniProt	MitoCarta3.0_SubMitoLocalization
Abcb6	ATP-binding cassette, sub-family B (MDR/TAP), member 6	Q9DC29	MOM
Acaa2	acetyl-Coenzyme A acyltransferase 2 (mitochondrial 3-oxoacyl-Coenzyme A thiolase)	Q8BWT1	Matrix
Acsf2	acyl-CoA synthetase family member 2	Q8VCW8	Matrix
Afg3l2	AFG3-like AAA ATPase 2	Q8JZQ2	MIM
Agxt2	alanine-glyoxylate aminotransferase 2	Q3UEG6	Matrix
Atpaf1	ATP synthase mitochondrial F1 complex assembly factor 1	H3BLL2	Matrix
Clpp	caseinolytic mitochondrial matrix peptidase proteolytic subunit	O88696	Matrix
Coq7	demethyl-Q 7	P97478	MIM
Cox5a	cytochrome c oxidase subunit 5A	P12787	MIM
Cox6c	cytochrome c oxidase subunit 6C	Q9CPQ1	MIM
Cyct	cytochrome c, testis	P00015	IMS
Dbt	dihydrolipoamide branched chain transacylase E2	P53395	Matrix
Dcakd	dephospho-CoA kinase domain containing	Q8BHC4	Membrane
Dglucy	D-glutamate cyclase	Q8BH86	Matrix
Echdc1	enoyl Coenzyme A hydratase domain containing 1	Q9D9V3	unknown
Echs1	enoyl Coenzyme A hydratase, short chain, 1, mitochondrial	Q8BH95	Matrix
Fam210a	family with sequence similarity 210, member A	Q8BGY7	MIM
Gpx4	glutathione peroxidase 4	Q91XR9	MIM
Hadha	hydroxyacyl-CoA dehydrogenase trifunctional multienzyme complex subunit alpha	Q8BMS1	MIM
Hsdl2	hydroxysteroid dehydrogenase like 2	Q2TPA8	Matrix
L2hgdh	L-2-hydroxyglutarate dehydrogenase	Q91YP0	MIM
Lactb2	lactamase, beta 2	Q99KR3	Matrix
Maob	monoamine oxidase B	Q8BW75	MOM
Mcee	methylmalonyl CoA epimerase	Q9D1I5	Matrix
Mff	mitochondrial fission factor	Q6PCP5	MOM
Mipep	mitochondrial intermediate peptidase	A6H611	Matrix
Mrpl18	mitochondrial ribosomal protein L18	Q9CQL5	Matrix
Mrpl20	mitochondrial ribosomal protein L20	Q9CQL4	Matrix
Mrpl45	mitochondrial ribosomal protein L45	Q9D0Q7	Matrix
Ndufa7	NADH:ubiquinone oxidoreductase subunit A7	Q9Z1P6	MIM
Ndufa9	NADH:ubiquinone oxidoreductase subunit A9	Q9DC69	MIM
Ndufb3	NADH:ubiquinone oxidoreductase subunit B3	Q9CQZ6	MIM
Ndufs4	NADH:ubiquinone oxidoreductase core subunit S4	Q9CXZ1	MIM
Ndufv3	NADH:ubiquinone oxidoreductase core subunit V3	Q8BK30	MIM
Oxsm	3-oxoacyl-ACP synthase, mitochondrial	Q9D404	Matrix
Pdk4	pyruvate dehydrogenase kinase, isoenzyme 4	O70571	MIM
Pmpcb	peptidase (mitochondrial processing) beta	Q9CXT8	Matrix
Ppox	protoporphyrinogen oxidase	P51175	MIM
Samm50	SAMM50 sorting and assembly machinery component	Q8BGH2	MOM
Slc25a42	solute carrier family 25, member 42	Q8R0Y8	MIM
Slc30a9	solute carrier family 30 (zinc transporter), member 9	Q5IRJ6	MIM
Snd1	staphylococcal nuclease and tudor domain containing 1	Q78PY7	unknown
Tmem186	transmembrane protein 186	Q9CR76	MIM
Tomm20	translocase of outer mitochondrial membrane 20	Q9DCC8	MOM
